# Changes in salivary markers during basketball long-term and short-term training periods: a systematic review

**DOI:** 10.5114/biolsport.2022.107018

**Published:** 2021-08-27

**Authors:** Paulius Kamarauskas, Daniele Conte

**Affiliations:** 1 Institute of Sport Science and Innovations, Lithuanian Sports University, Kaunas, Lithuania

**Keywords:** Hormonal response, Testosterone, Cortisol, Physiological demand, Strength training

## Abstract

Changes in salivary markers have been largely assessed during different modalities of long-term and short-term basketball training across different basketball populations. The aim of this paper was to systematically review the literature assessing changes in salivary markers in basketball following long-term and short-term training periods. An electronic database search of articles published until October 2020 was completed in PubMed, SPORTDiscus, Scopus and Web of Science. Studies were then screened and selected using pre-defined selection criteria with 1080 articles identified. After removing 690 duplicates, 390 articles were included for screening, which revealed 15 articles that met the inclusion criteria. The main findings revealed no changes in testosterone (T), cortisol (C) or their ratio (T:C), while contrasting results were found in immunoglobulin A (IgA) and total protein (TP) levels across long-term periodized training periods in different basketball populations. The analysis of short-term training periods showed that strength-hypertrophy training induced higher C levels compared to a non-exercising day, one-power training and one-endurance training session in female basketball players, while no changes were evident for T and IgA. Moreover, the analysis of salivary markers in response to small-sided games (SSGs) documented a large-to-moderate increase in alpha-amylase (AA) from pre- to post-SSG and inconsistent results of C and T across differently designed SSGs. The current results provide a detailed description of salivary marker changes in response to different basketball long- and short-term training periods, which can help practitioners in designing sound training programmes to optimize players’ fitness and health status across different phases of the season.

## INTRODUCTION

Basketball is considered as a contact, intensive and dynamic sport in which the athlete’s performance depends on physical demands (i.e. power, speed, agility, endurance [1]) and physiological responses (i.e. heart-rate response, lactate concentration, oxygen consumption [1–3]). Additionally, social (e.g. relationships, living conditions, microclimate in the team) and psychological (e.g. mood, motivation) factors play an important role in determining the level of performance [4]. In fact, basketball matches require athletes to repeat maximal efforts in offensive and defensive phases with short rest periods in between [5]. Moreover, the schedule of the basketball season for teams competing at a high level (i.e. semi-professional, professional) might be characterized by a congested match schedule, which could induce high fatigue and low readiness to play [3, 6–8]. Therefore, monitoring the workload imposed by basketball training and matches is fundamental to monitor fatigue, identifying injury risk, and determining player readiness to perform [8–11].

Workload has been classified as external load, which is considered the physical load encountered by players (i.e. stimulus imposed), and internal load, which is considered the biochemical, physiological and psychological responses induced by training or matches [12, 13]. The increased availability of modern technologies to monitor external load (i.e. the Global Positioning System [GPS] or Local Positioning Systems [LPS]) has downsized the attention given for internal load or actual psychophysiological responses [14]. However, the importance of monitoring the internal responses to the imposed stimuli has recently been emphasized to provide a full picture of the athlete’s status [14]. In this regard, salivary markers such as testosterone (T), cortisol (C) and immunoglobulin A (IgA) are among the most adopted tools to monitor hormonal responses to given loads in team sports and particularly in basketball [15–17]. Specifically, T and C are widely used to measure the balance between anabolic and catabolic processes [9, 18–20]; for the detection of overtraining and overreaching [21, 22]; for the assessment of psychophysiological factors such as stress levels derived from training and matches [23, 24]; to track athletes’ recovery processes [21, 25]; and to avoid a possible decline in performance [25, 26]. Furthermore, since performance strongly depends on the overall wellness and health status of athletes [11], illnesses such as infections can be the reasons for disrupted ability to prepare and perform well [27]. In this regard, monitoring salivary IgA, which is an oral mucosal immune marker suggested to provide information about upper respiratory tract infection (URTI) [22], and to provide the first line of defence against pathogens and antigens due to its predominance in mucosal secretions [28], has been considered essential [27]. Considering the benefits of monitoring biological and physiological responses during the training process [10], the recommendation to use salivary markers has been previously emphasized [29, 30]. Indeed, salivary analysis have been shown to have some medical and practical advantages. From a medical standpoint, saliva collection is a non-invasive method allowing to reduce the risk of possible infections compared to other methods such as blood analysis [16, 31]. From a practical standpoint, the advantages of using salivary markers are an overall lower cost and acceptability by the athletes compared to invasive methods [31].

During the last decade, the body of literature assessing salivary markers in basketball research has consistently grown [15, 16, 19, 21, 22, 23, 26, 32, 33, 34, 35, 36, 37, 38, 39, 40, 41]. The analysis of salivary responses was implemented in different ages and levels of competition during training sessions [15, 16, 19, 21–23, 36, 41, 42] and within different phases of the season [26, 40]. However, to the best of our knowledge, there is not a systematic revision of the literature assessing the changes in salivary responses during basketball training. Therefore, the aim of this systematic review is to synthesize findings about salivary markers adopted during basketball training.

## MATERIALS AND METHODS

This systematic review was conducted following the Preferred Reporting Items for Systematic Reviews and Meta-Analyses (PRISMA) guidelines [43]. The protocol of the systematic review was not registered at inception since this process is not mandatory to conduct a systematic review [44].

### Literature search strategy

The search strategy presented in Table 1 was used for the identification of articles in four electronic databases (PubMed, SPORTDiscus, Scopus and Web of Science). Articles published online or in-print from database inception until October 2020, when the search was conducted for the last time, were included. Three search variables (Salivary markers & Type of activity & Basketball) were used in all possible combinations for the search strategy. Only original peer-reviewed articles published in English were considered while other type of publications (e.g. literature reviews, conference proceedings) were excluded.

**TABLE 1:** Search strategy used to locate relevant research articles.

	Variable	Search terms
1.	Salivary markers	(¢hormonal response*¢ OR ¢salivary cortisol¢ OR ¢salivary testosterone¢ OR ¢salivary immunoglobulin A¢ OR ¢salivary marker*¢ OR ¢endocrinology¢)
2.	Type of activity	(¢training*¢)
3.	Basketball	(¢basketball¢)
Salivary markers AND type of activity AND basketball		¢1 AND 2 AND 3¢

### Selection criteria

Preferred Reporting Items for Systematic Reviews and Meta-Analyses (PRISMA) guidelines [43] were followed during the article screening process. Articles consisting of the analysis of salivary marker changes during basketball training were included in the review. Selectio n criteria were created and used without any restrictions for study samples (e.g. age, sex, playing level, or playing experience), or study designs (e.g. cross-sectional, longitudinal, experimental).

### Study categorization

For the purpose of this review, studies were categorized in two sections:

––Long-term effect of basketball training periods (i.e. periodized training programmes, different phases of the season) on salivary marker levels;
––Effect of short-term basketball training sessions (i.e. small-sided games) on salivary marker levels

**FIG. 1:**
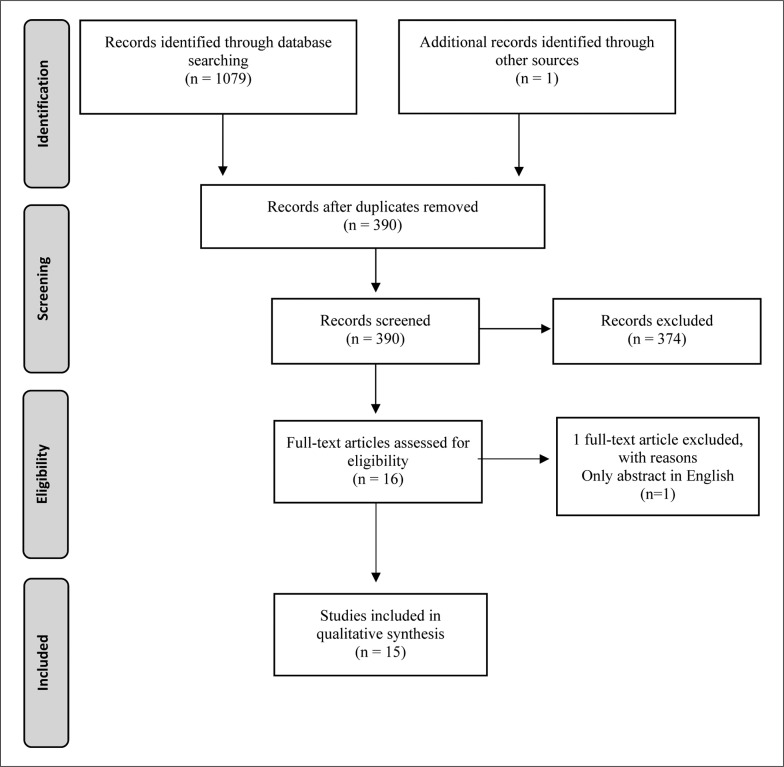
Preferred Reporting Items for Systematic Reviews and Meta-Analysis (PRISMA) flow diagram of search strategy.

The literature search led to the identification of the following salivary markers: C, T, their ratio (T:C), IgA, lactoferrin (LF), alpha-amylase (AA), total protein (TP), IgA-to-TP ratio (IgA:TP). After exclusion of duplicates, the abstracts of all identified articles were screened independently against the pre-defined selection criteria by two authors (PK and DC). The same screening process was then applied for the full-text version of the included articles. Additionally, the reference list of each included article was then hand-searched with one relevant article included during the identification process (Figure 1). This type of search strategy has been used in other systematic reviews [1, 45, 46].

### Procedures

#### Assessment of methodological quality

The modified version of the Downs and Black checklist for assessment of methodological quality of randomised and non-randomised healthcare interventions [47] was used to assess the methodological quality of included articles. The checklist was chosen as the validation of the method was proved [47] and the checklist has been previously used to assess methodological quality in systematic reviews [48–50]. The number of items from the original checklist can be adjusted to the scope and need of the systematic review, with 10 to 15 items utilized in previous systematic reviews [48–50]. For this review, the checklist was combined for non-interventional and for interventional study designs, respectively of the 12 and 13 most relevant items, which are presented in Table 2. Each item is scored as 1 = “Yes”, and 0 = “No/unable to determine”. The scores for each of the 12 or 13 items were summed to provide the total quality score. The quality of each included article was independently evaluated by two authors (PK and DC).

**TABLE 2:** Questions of the modified Downs and Black checklist used for the assessment of methodological quality of the included articles.

	Question
**No.**	**Reporting**

1	Is the hypothesis/aim/objective of the study clearly described?
2	Are the main outcomes to be measured clearly described in the Introduction or Methods section?
3	Are the characteristics of the patients/subjects included in the study clearly described?
4	Are the main findings of the study clearly described?
5	Does the study provide estimates of the random variability in the data for the main outcomes?
6	Have actual probability values been reported (e.g. 0.035 rather than < 0.05) for the main outcomes except when the probability value is less than 0.001?

**External validity**	
7	Were the subjects asked to participate in the study representative of the entire population from which they were recruited?
8	Were those subjects who were prepared to participate representative of the entire population from which they were recruited?
**Internal validity**	

9	If any of the results of the study were based on “data dredging”, was this made clear?
10	In trials and cohort studies, do the analyses adjust for different lengths of follow-up of patients, or in case control studies, is the time period between the intervention and outcome the same for cases and controls?
11	Were the statistical tests used to assess the main outcomes appropriate?
12	Were the main outcome measures used accurate (valid and reliable)?
13	Was compliance with the intervention/s reliable? (*Just for interventional studies)*

#### Data extraction and analysis

For the identification and extraction of representative data, all included articles were analysed by the lead author (PK). Data not provided or presented non-numerically were identified as “not reported”. During the identification process, if provided, the following data were extracted and presented in tables:

––Characteristics of participants: sample size, playing level, sex, age, stature and body mass;
––Research methodology: selection of salivary markers, use of saliva flow rate stimulation, use of mouth rinse before collection, dietary restrictions due to saliva collection, collection type (i.e. passive drooling, swabbing, spitting), time of the collection, manufacturer of reagents used for analysis;
––Methodological outcome measures: phase of the season, duration of monitoring period, type of activities monitored, frequency of saliva sample collection, salivary markers analysed and variability in results of analysis of salivary markers;
––Study results: outcomes of saliva analysis (i.e. differences, statistical significances, effect sizes and interpretation).

Where possible, participants’ characteristics are reported as mean ± standard deviation (SD). The type of methodology used to collect saliva samples are presented in Table 3.

**TABLE 3:** Types of methodology used to collect saliva samples in the included articles.

Study	Salivary markers	Stimulated Yes / No	Mouth rinse Yes / No	Dietary restriction time	Collection type	Time of the collection	Manufacturer of reagents
**Long-term effect of basketball training periods on salivary marker levels**							
Andre et al. 2018 [26]	Cortisol Testosterone	No	Yes	60 min	Swabbing	1:00–3:00pm	Salimetrics
Arruda et al. 2013 [23]	Cortisol	No	No	Overnight fasting	n/a	7:30am	DSL
Atalag et al. 2019 [32]	Cortisol	No	No	60 min	Passive drooling	10:00am–12:00pm	Salimetrics
Gonzalez-Bono et al. 2002 [51]	Cortisol Testosterone	No	No	Overnight fasting	Passive drooling	8:30–9:00am	OD (C) ICN (T)
He et al. 2010 [25]	Cortisol Immunoglobulin A Total protein Lactoferrin	No	Yes	n/a	Spitting	5:30–6:30pm	DRG (C) Sigma (IgA) Calbiochem (LF)
Moreira et al.2011 [40]	Cortisol Immunoglobulin A	No	No	60–90 min	Passive drooling	n/a (in the afternoon, same time of the day)	ALPCO
Nunes et al. 2011(a) [15]	Cortisol Testosterone Immunoglobulin A	No	No	n/a	Passive drooling	7:30am; 9:30am; 11:00am; 5:30pm	Salimetrics (T; IgA) DSL (C)
Nunes et al. 2014 [18]	Cortisol Testosterone Immunoglobulin A	No	Yes	90 min	Passive drooling	7:30am; 9:30am; 12:00pm; 6:00pm	Salimetrics
Miloski et al. 2015 [36]	Testosterone	No	No	120 min	Passive drooling	3:00pm	Salimetrics
Azarbayjani et al. 2011 [21]	Immunoglobulin A Total protein	No	Yes	n/a	n/a	n/a (pre-, post- and 1h post- completion of exercise)	Bradford
Moraes et al. 2017 [22]	Immunoglobulin A	No	No	120 min	Passive drooling	3:00pm	Salimetrics
Moreira et al. 2008 [42]	Immunoglobulin A Total protein	No	Yes	120 min	Passive drooling	3:30pm	ALPCO (IgA) Pierce (TP)
**Short-term effect of basketball training periods on salivary marker levels**							
Moreira et al. 2018 [19]	Cortisol Testosterone Alpha-amylase	No	No	n/a	n/a	n/a (pre- control and experimental conditions; post- both conditions; post- SSGs)	Salimetrics
Nunes et al. 2011(b) [41]	Cortisol Testosterone Immunoglobulin A	No	No	n/a	n/a	7:30am; 9:30am; 11:00am; 5:30pm	Salimetrics
Sansone et al. 2018 [16]	Cortisol Testosterone	No	No	n/a	n/a	n/a (pre- warm-up, and 15min post- SSGs. Experimental sessions started at 5:00pm)	LDN

Note: n/a – not available, not provided in article; SSGs – small-sided games; Salimetrics – Salimetrics LLC, Carlsbad, CA, USA; DSL – Diagnostic Systems Laboratories, INC, Webster, TX, USA; ICN – ICN Biomedicals, Costa Mesa, CA, USA; OD – Orion Diagnostica, Espoo, Finland; DRG – DRG Diagnostics, Marburg, Germany; Sigma – Sigma-Aldrich, Poole, UK; Calbiochem – Calbiochem, Darmstadt, Germany; ALPCO – ALPCO diagnostics, Salem, MA, USA; Bradford – Bradford Solution for Protein Determination; Pierce – Pierce Biotechnology, Rockford, IL, USA; LDN – Labor Diagnostika Nord, Germany; C – cortisol; T – testosterone; IgA – immunoglobulin A; AA – alpha-amylase; TP – total protein; LF – lactoferrin.

## RESULTS

### Search findings and study selection

Through the electronic database search, 1079 articles were found (PubMed = 231, SPORTDiscus = 162, Scopus = 277, Web of Science = 409). An additional article was found as potentially relevant through the search of other sources for a total of 1080 identified articles. After removing 690 duplicate records, 390 records were included for a further analysis of eligibility. Screening of titles and abstracts led to the removal of a further 374 articles based on title and abstract before the full-text screening procedure. The full-text eligibility check comprised 16 articles being screened with 1 article removed from the analysis since only the abstract was written in English. Fifteen articles matched all the selection and evaluation criteria and were included in this systematic review. The full results of the search are presented in Figure 1.

### Methodological quality

The results of methodological quality evaluation for each included article are presented in Table 4. The total scores range from 7 to 10 for non-interventional studies (maximum possible score = 12) and from 7 to 12 for interventional studies (maximum possible score = 13). Similarly to other systematic reviews that used the Downs and Black checklist [45, 48–50], no articles were excluded based on the results of methodological quality evaluation.

**TABLE 4:** Results of methodological quality assessment for included articles.

Study	Downs and Black checklist question number													TOTAL
	Reporting						External validity		Internal validity-bias					
	1	2	3	4	5	6	7	8	9	10	11	12	13	
**Long-term effect of basketball training periods on salivary marker levels**														
Andre et al. 2018 [26]	1	1	0	1	1	1	0	1	1	1	1	1	T	10
Arruda et al. 2013 [23]	1	1	1	0	0	0	0	1	1	0	1	1	T	7
Atalag et al. 2019 [32]	1	1	1	1	1	0	0	1	1	1	1	1	T	10
Gonzalez-Bono et al. 2002 [51]	1	1	1	1	1	1	0	1	1	1	1	1	1	12
He et al. 2010 [25]	1	1	0	1	1	0	0	1	1	1	1	1	T	9
Moreira et al. 2011 [40]	1	1	1	1	1	0	0	0	1	1	1	1	T	9
Nunes et al. 2011(a) [15]	1	1	0	0	0	0	0	0	1	1	1	1	1	7
Nunes et al. 2014 [18]	1	1	1	0	0	0	0	1	1	1	1	1	1	9
Miloski et al. 2015 [36]	1	1	1	1	1	1	0	1	1	1	1	1	1	12
Azarbayjani et al. 2011 [21]	1	1	1	1	1	0	0	0	1	1	1	1	1	10
Moraes et al. 2017 [22]	1	1	1	0	0	1	0	1	1	1	1	1	1	10
Moreira et al. 2008 [42]	1	1	1	1	1	0	0	1	1	1	1	1	T	10

**Short-term effect of basketball training periods on salivary marker levels**														
Moreira et al. 2018 [19]	1	1	1	0	0	0	0	1	1	1	1	1	1	9
Nunes et al. 2011(b) [41]	1	1	1	0	0	0	0	1	1	1	1	1	1	9
Sansone et al. 2018 [16]	1	1	1	1	1	1	0	1	1	1	1	1	1	12

Note: 1 = Yes; 0 = No/Unable to determine; T – non-interventional study.

### Participant characteristics

The characteristics of the participants assessed in the included articles are presented in Table 5. Studies investigating samples of different sizes, ranging from 8 to 36 participants, were included in the final analysis. Analysis of salivary markers in basketball was performed for both male and female participants with 11 articles including males only, and 4 articles including only female players. Participants from included articles were competing in different basketball levels and age categories: youth (n = 2), sub-elite collegiate (n = 2), elite collegiate (n = 1), amateur (n = 1), sub-elite (n = 1) and elite basketball (n = 8).

**TABLE 5:** Characteristics of the participants in included articles.

Study	Sample size (N) Final (Initial)	Level	Sex	Age (years)(mean ± SD)	Stature (cm)(mean ± SD)	Body mass (kg)(mean ± SD)
**Long-term effect of basketball training periods on salivary marker levels**						
Andre et al. 2018 [26]	12	Elite Collegiate	Male	n/a	n/a	n/a
Arruda et al. 2013 [23]	12	Elite	Female	26.2 ± 3.9	183.1 ± 9.8	74.5 ± 10.1
Atalag et al. 2019 [32]	Basketball players: 36	Sub-Elite Collegiate	Male	Basketball players 21.32 ± 1.7	Basketball players 191.43 ± 9.02	Basketball players98.99 ± 16.15
Gonzalez-Bono et al.2002 [51]	18 Team 1 (T1) (N = 10) Team 2 (T2) (N = 8)	Elite	Male	T1: 21.60 ± 1.07 # T2: 21.50 ± 1.69 #	T1: 195 ± 0.02 # T2: 195 ± 0.03 #	T1: 90.79 ± 3.73 # T2: 93.56 ± 3.34 #
He et al. 2010 [25]	8	Sub-Elite Collegiate	Male	20.5 ± 0.3 #	176.6 ± 2.0 #	75.1 ± 3.9 #
Moreira et al. 2011 [40]	15	Elite	Male	19 ± 0.6	192 ± 10	92 ± 9
Nunes et al. 2011(a) [15]	12	Elite	Female	26.2 ± 3.9	183.1 ± 9.8	82.2 ± 13.1
Nunes et al. 2014 [18]	19	Elite	Female	26 ± 5	181.8 ± 7.2	75.6 ± 12.6
Miloski et al. 2015 [36]	16 (23)	Brazilian state youth	Male	15.3 ± 0.7*	186.1 ± 8.9*	82.4 ± 14.6*
Azarbayjani et al. 2011 [21]	20	Amateur	Male	24.4 ± 3.6	184 ± 10	83.5 ± 3.6
Moraes et al. 2017 [22]	23	Brazilian state youth	Male	15.8 ± 0.8	n/a	82.7 ± 13.0
Moreira et al. 2008 [42]	10Basketball players (B) (N = 5) Coaching staff (C) (N = 5)	Elite	Male	B: 23 ± 2 C: 40 ± 6	B: 206 ± 4 C: 177 ± 5	B: 116 ± 11 C: 83 ± 7
**Short-term effect of basketball training periods on salivary marker levels**						
Moreira et al. 2018 [19]	32 [48] U14 (N = 14) U15 (N = 10) U16 (N = 8)	Elite	Male	15.2 ± 1.2	180 ± 11	72 ± 15
Nunes et al. 2011(b) [41]	14	Elite	Female	26.2 ± 3.9	183.1 ± 9.8	74.5 ± 10.1
Sansone et al. 2018 [16]	12	Sub-Elite	Male	21 ± 2	193.9 ± 7.0	84.8 ± 6.6

Note: n/a – not provided;

* – average data reported for initial sample size; SD – standard deviation.

# – data reported as mean ± SEM (standard error of the mean).

### Outcome measures

Outcome measures of included articles are presented in Table 6. Different markers were used across the identified articles with C, T and IgA being the most studied markers: C (n = 11), T (n = 8), T:C (n = 3), IgA (n = 8), TP (n = 3), IgA:TP (n = 2), LF (n = 1), AA (n = 1). Dependently on the purpose of each study, saliva samples were collected at different times of the day, with different gaps between collections and in some cases at additional collection points (i.e. after rest or recovery periods). The most adopted type of collection is pre- to post-activity (training session, microcycle, small-sided game, preparation or competitive period, training programme etc.). In the identified studies, we also considered whether the coefficient of variation values (CVs) were reported for intra- and inter-assay, which are typical analyses used to verify the reliability of measurements. The obtained intra-assay and inter-assay CVs are displayed in Table 6. CVs were reported in: i) 9 (intra-assay; range: 2.5% – 5.2%) and 5 (inter-assay; range: 3.6% – 7.8%) articles out of 11 C articles; ii); 8 (intra-assay; range: 3.7% – 5.5%) and 4 (inter-assay; range: 4.2% – 6.9%) articles out of 8 T studies; iii) 5 (intra-assay; range: 3.0% to 6.0%) and 2 (inter-assay; exact value = 9.1%) articles out of 8 IgA articles. For other salivary markers, CVs were reported only for intra-assay with values of 2.6% for AA and 4.0% for LF with no CV reported for TP.

**TABLE 6:** Methodological outcome measures of included articles

Study	Duration	Type of activity	Frequency of saliva collection	Salivary markers	Coefficient of variation forthe assays (%)
**Long-term effect of basketball training periods on salivary marker levels**					
Andre et al. 2018 [26]	30 weeks	Pre-season and in-season of NCAA Division I	Weekly	CT	C = 3.2 intra / 3.6 inter T = 3.7 intra / 4.2 inter
Arruda et al. 2013 [23]	5 weeks	Three preparation microcycles encom-passing 1 week of muscular endurance, 2 weeks of strength, 2 weeks of power	At the beginning and after each microcycle.	C	Inter-assay between 2.5 and 7.8
Atalag et al. 2019 [32]	Season	In-season phase of student basketball league	Pre- to post-in-season phase	C	< 7.0 intra & inter
Gonzalez-Bono et al. 2002 [51]	4 months	Two periodical sessions of maximal cycle ergometer test were carried out 4 months apart.	Pre- to post-periodical sessions.	C T	< 5.0 intra & inter
He et al. 2010 [25]	11 weeks	11-week period consisting of 4-week preparation, 3-week of competition (2^nd^ week for rest) and 4-week recovery period	Pre- to post-preparation and recovery periods and in the middle of competitive weeks.	C IgA TP LF	C = 4.0 intra IgA = 3.0 intra LF = 4.0 intra
Moreira et al. 2011 [40]	4 weeks	28 days of regular training during the in-season phase, playing one weekly match.	Pre- to post-experimental period.	C IgA	n/a
Nunes et al. 2011(a) [15]	7 weeks	Three periodized cycles encompassing 3 weeks of muscular endurance, 2 weeks of strength and 2 weeks of power.	Pre- to post-preparation and after 2 days of rest.	C T IgA	C = 2.5 intra / 7.8 inter T = 3.7 intra / 6.9 inter IgA = 4.2 intra / 9.1 inter
Nunes et al. 2014 [18]	12 weeks	A 12-week preparation period including 2 overloading periods (weeks 4–6 and 8–10) followed by 1-week and 2-week tapering periods, respectively.	Pre- to post-training programme at 7:30, 9:30, 12:00 and 18:00.	C T IgA	C = 5.2 intra T = 4.5 intra IgA = 3.8 intra
Miloski et al. 2015 [36]	8 weeks	1 week of familiarization and 4 weeks of overloading followed by 3 weeks of tapering phase.	Pre- to post-overloading and post tapering phase.	T	5.3 intra
Azarbayjani et al. 2011 [21]	8 weeks	Progressive exercise training on the treadmill, consisting of interval and continuous parts 3 bouts per week.	Before, immediately and 1 hour after each training bout.	IgA TP	n/a
Moraes et al. 2017 [22]	8 weeks	1 week of familiarization and 4 weeks of overloading followed by 3 weeks of tapering phase.	Pre- to post-overloading and post tapering phase.	IgA	6.0 intra
Moreira et al. 2008 [42]	17 days	Preparation period consisting of technical, tactical, strength and conditioning sessions.	Pre- to post-preparation.	IgA TP	n/a
**Short-term effect of basketball training periods on salivary marker levels**					
Moreira et al. 2018 [19]	1 week	Two SSG 4 × 4 with control (cognitive) and experimental (mental fatigue) procedures before.	Pre- to post-control and experimental trials, and post SSGs.	C T AA	C = 4.4 intra T = 4.6 intraAA = 2.6 intra
Nunes et al. 2011(b) [41]	5 weeks	Control and 3 experimental (endurance, strength and power training) sessions over a period of 40 days. Experimental sessions were separated by 14 days.	On Control day at 7:30, 9:30, 11:00, 17:30. On experimental days pre-to-post exercise and at 17:30.	C T IgA	C = 2.5 intra / 7.8 inter T = 3.7 intra / 6.9 inter IgA = 4.2 intra / 9.1 inter
Sansone et al. 2018 [16]	4 weeks	Four sessions of SSG 3 × 3 with different tactical tasks and training regimes.	Pre- to post-SSGs.	C T	C = 4.2 intra T = 5.5 intra

Note: n/a – not available and not provided in article; SSG – small-sided game; C – cortisol; T – testosterone; IgA – immunoglobulin A; AA – alpha-amylase; TP – total protein; LF – lactoferrin.

### Salivary markers’ responses to long-term training periods

Twelve articles investigated changes in salivary markers following a long-term training period [15, 18, 21, 22, 23, 25, 26, 32, 36, 40, 42, 51] (Table 7).

**TABLE 7:** Long-term effect of basketball training periods on salivary marker levels

Study	Marker	Measures	Level (mean ± SD)	Changes
Andre et al. 2018 [26]	C	Overall change during the season	Season mean = 9.1 nmol/l	p < 0.001
		Beginning of pre-season (week 2)	7.4 ± 2.7 nmol/l	p < 0.05
		Before start of regular season (week 6)	6.9 ± 2.7 nmol/l	p < 0.01
		Start of the season (week 7)	5.5 ± 3.3 nmol/l	p < 0.01
		In-season (week 10)	6.9 ± 2.6 nmol/l	p < 0.01
		After the holiday break (week 15)	13.1 ± 5.8 nmol/l	p < 0.05
		Beginning of important matches (week 17)	20.3 ± 8.9 nmol/l	p < 0.01
		In-season (week 22)	6.9 ± 2.8 nmol/l	p < 0.05
		End of regular season (week 24)	6.9 ± 2.1 nmol/l	p < 0.01
		Post-season (week 27)	6.4 ± 2.2 nmol/l	p < 0.01
		Post-season (week 28)	17.7 ± 12.6 nmol/l	p < 0.05
		Post-season (week 30)	7.1 ± 3.1 nmol/l	p < 0.05
	T	Overall change of testosterone during the season	Season mean = 0.51 nmol/l	p < 0.001
		In-season (week 13)	0.58 ± 0.15 nmol/l	p < 0.05
		In-season after the holiday break (week 15)	0.59 ± 0.10 nmol/l	p < 0.05
		In-season (week 16)	0.37 ± 0.08 nmol/l	p < 0.01
		Beginning of important matches (week 17)	0.69 ± 0.25 nmol/l	p < 0.05
		In-season (week 21)	0.40 ± 0.08 nmol/l	p < 0.01
		In-season (week 22)	0.40 ± 0.08l nmol/l	p < 0.05
		End of regular season (week 24)	0.39 ± 0.06 nmol/l	p < 0.01
		One week before the conference tournament (week 25)	0.41 ± 0.05 nmol/l	p < 0.01
		End of the season (week 27)	0.43 ± 0.07 nmol/l	p < 0.05
		Post-season (week 28)	0.62 ± 0.14 nmol/l	p < 0.05
	T:C	Season mean T:C ratio	Season mean for T:C = 0.069	
		Start of the regular season (week 7)	0.110 ± 0.50	p < 0.01
		Beginning of important matches (week 17)	0.041 ± 0.25	p < 0.01
		One week before the conference tournament (week 25)	0.056 ± 0.17	p < 0.01
Arruda et al. 2013 [23]	C	Pre- to post-microcycles	Not provided	p > 0.05
Atalag et al. 2019 [32]	C	Pre- to post-in-season phase	Pre: 5.7 ± 2.2 nmol/l Post: 13.2 ± 6.7 nmol/l	p < 0.01
Gonzalez-Bono al. 2002 [51]	C	Comparison of Team 1 and Team 2 at baseline, pre-first periodical session	Team 1: 5.64 ± 0.95 nmol/l * Team 2: 3.10 ± 0.54 nmol/l *	p < 0.05
		Comparison of post-ergometry concentrations after the first periodical session	Team 1: 4.74 ± 0.92 nmol/l * Team 2: 6.1 ± 1.23 nmol/l *	p < 0.02
		Comparison of post-ergometry concentrations after the second periodical session	Team 1: 3.24 ± 0.78 nmol/l *	
			Team 2: 4.02 ± 0.79 nmol/l *	p < 0.05
		Effect of training programme on response to the cycle ergometry periodical sessions for Team 1	1^st^ session: 4.74 ± 0.92 nmol/l * 2^nd^ session: 3.24 ± 0.78 nmol/l *	p > 0.05
		Effect of training programme on response to the cycle ergometry periodical sessions for Team 2	1^st^ session: 6.1 ± 1.23 nmol/l * 2^nd^ session: 4.02 ± 0.79 nmol/l *	p < 0.05
	T	Comparison of Team 1 and Team 2 at baseline, pre-first periodical session	Team 1: 200.15 ± 31.87 pmol/l * Team 2: 179.12 ± 34.36 pmol/l *	p > 0.05
		Comparison of post-ergometry concentrations after the first periodical session	Team 1: 252.50 ± 56.04 pmol/l * Team 2: 235.51 ± 41.79 pmol/l *	p > 0.05
		Comparison of post-ergometry concentrations after the second periodical session	Team 1: 151.38 ± 19.69 pmol/l * Team 2: 164.28 ± 23.86 pmol/l *	p > 0.05
		Effect of training programme on response to the cycle ergometry periodical sessions for Team 1	Session 1: 252.50 ± 56.04 pmol/l * Session 2: 151.38 ± 19.69 pmol/l *	p > 0.05
		Effect of training programme on response to the cycle ergometry periodical sessions for Team 2	Session 1: 235.51 ± 41.79 pmol/l * Session 2: 164.28 ± 23.86 pmol/l *	p > 0.05
	T:C	Interaction between factors TEAM and SESSION	Team 1 pre-first test: 0.042 ± 0.009 Team 1 post-first test: 0.065 ± 0.016 Team 2 pre-first test: 0.066 ± 0.015 Team 2 post-first test: 0.046 ± 0.011 Team 1 pre-second test: 0.074 ± 0.016 Team 1 post-second test: 0.060 ± 0.013 Team 2 pre-second test: 0.045 ± 0.013 Team 2 post-second test: 0.055 ± 0.013	p < 0.01
		Effect of training programme for Team 1		p < 0.06
		Effect of training programme for Team 2		p < 0.07
		Effect of factor TEAM for Team 1		p < 0.02
		Effect of factor TEAM for Team 2		p < 0.01
He et al. 2010 [25]	C	Pre- to post- (T4-to-R4) (4-week preparation, 3-week competition & 4-week recovery period)	T4: 71.0 ± 2.2 ng/ml T1: 48.0 ± 4.9 ng/ml C1: 63.6 ± 4.1 ng/ml M1: 46.6 ± 4.5 ng/ml C2: 84.4 ± 4.1 ng/ml R1: 47.2 ± 4.0 ng/ml R4: 40.6 ± 3.9 ng/ml	p < 0.01
		Changes from week 4 (T1) of preparation to post-recovery (R4)		p < 0.05
		Changes from pre-competition-to-post-recovery (C1-to-R4)		p < 0.05
		Changes from week of rest between competition weeks (M1) to the end of 4-week recovery period (R4)		p > 0.05
		Changes from week 2 of competition (C2) to the end of 4-week recovery period (R4)		p < 0.01
		Changes from pre- to post- (R1-to-R4) 4-week recovery period		p < 0.05
	Secretion rate C	Week 1 of training vs. Week 4 of recovery	Higher than at week 4 of recovery	p < 0.01
		Week 4 of training vs. Week 4 of recovery		p < 0.05
		Week 1 of competition vs. Week 4 of recovery		p < 0.05
		Week 2 of competition vs. Week 4 of recovery		p < 0.01
		Pre- to post-recovery weeks		p < 0.05
	IgA	Pre- to post- (T4-to-R4) (4-week preparation, 3-week competition & 4-week recovery)	T4: 146.7 ± 18.0 ug/ml T1: 144.9 ± 22.7 ug/ml C1: 142.9 ± 11.9 ug/ml M1: 204.9 ± 9.5 ug/ml C2: 153.2 ± 18.0 ug/ml R1: 204.3 ± 20.5 ug/ml R4: 210.7 ± 15.0 ug/ml	p < 0.01
		Changes from week 4 (T1) of preparation to post-recovery (R4)		p < 0.05
		Changes from week 1 (C1) of competition to post-recovery (R4)		p < 0.01
		Changes from week 2 (C2) of competition to post-recovery (R4)		p < 0.05
		Changes from week of rest between competition weeks (M1) to the end of 4-week recovery period (R4)		p > 0.05
		Pre- to post-4-week recovery period		p > 0.05
	Secretion rate IgA	Week 1 of training vs. Week 4 of recovery	Not provided (Lower compared with week 4 of recovery)	p < 0.01
		Week 4 of training vs. Week 4 of recovery		p < 0.05
		Week 1 of competition vs. Week 4 of recovery		p < 0.01
		Week 2 of competition vs. Week 4 of recovery		p < 0.05
	TP	Absolute concentrations of salivary total protein measure at different time points	T4: 1109.5 ± 192.0 ug/ml T1: 815.7 ± 139.4 ug/ml C1: 1254.5 ± 355.6 ug/ml M1: 964.6 ± 141.3 ug/ml C2: 877.6 ± 288.8 ug/ml R1: 1434 ± 362.6 ug/ml R4: 1141.6 ± 191.7 ug/ml	p > 0.05
	Secretion rate TP	Secretion at different time points		p > 0.05
	LF	Changes from pre- to post- (T4-to-R4) (4-week preparation, 3-week competition & 4-week recovery)	T4: 3247.1 ± 635.7 ug/ml T1: 3440.8 ± 739.1 ug/ml C1: 2634.4 ± 546.9 ug/ml M1: 2728.6 ± 441.6 ug/ml C2: 3684.1 ± 602.7 ug/ml R1: 4619.8 ± 819.7 ug/ml R4: 4300.8 ± 905.3 ug/ml	p < 0.05
		Changes from week 4 (T1) of preparation to post-recovery (R4)		p < 0.05
		Changes from week 1 (C1) of competition to post-recovery (R4)		p < 0.05
		Changes from week 2 (C2) of competition to post-recovery (R4)		p < 0.05
		Changes from week of rest between competition weeks (M1) to the end of 4-week recovery period (R4)		p > 0.05
		Pre- to post-4-week recovery period (R1-to-R4)		p > 0.05
	Secretion rate LF	Week 1 of training vs. Week 4 of recovery	Not provided (Lower compared with week 4 of recovery)	p < 0.05
		Week 4 of training vs. Week 4 of recovery		p < 0.05
		Week 1 of competition vs. Week 4 of recovery		p < 0.05
		Week of rest between competition vs. Week 4 of recovery		p < 0.05
Moreira et al. 2011 [40]	C	Pre- to post-1-month of in-season phase	Pre: 17.6 ± 1.8 ng/ml * Post: 26.8 ± 4.9 ng/ml *	p < 0.05
	IgA		Pre: 587 ± 94 ug/ml * Post 720 ± 153 ug/ml *	p > 0.05
	Secretion rate IgA		Pre: 106 ± 20 ug/min * Post: 92 ± 21 ug/min *	p < 0.05
	Nunes et al. 2011(a) [15]				C	Pre- to post-preparation	Not provided	p > 0.05
Time of the day effect (07:30, 9:30, 11:00, 17:30) pre-to-post preparation		Not provided (Significant increase at 9:30 during both the pre- and post-training assessments)	p < 0.05		
T		Pre- to post-preparation	Not provided	p > 0.05
		Time of the day effect (07:30, 9:30, 11:00, 17:30) pre-to-post preparation		p > 0.05
T:C		Comparison of T:C from pre-to-post training at 4 sampling points (7:30, 9:30, 11:00, 17:30)	Not provided (T:C increased from pre-to-post training at 7:30)	p < 0.05
IgA		Time of the day effect (07:30, 9:30, 11:00, 17:30) pre-to-post preparation	Not provided	p > 0.05
		Pre- to post-training programme at 9:30		p < 0.05
		Pre- to post-training programme at 11:00		p < 0.05
Nunes et al. 2014 [18]	C	1 day pre-to-1-day post 12-week training at 4 sampling points (7:30, 9:30, 12:00 and 18:00)	Not provided	p > 0.05
	T	Pre- to post-training at 4 sampling points (7:30, 9:30, 12:00, 18:00)		p > 0.05
	IgA	Pre- to post-training at 4 sampling points (7:30, 9:30, 12:00, 18:00)		p > 0.05
Miloski et al. 2015 [36]	T	High testosterone concentration group (HTC) vs. Low testosterone concentration group (LTC) at baseline	HTC: 529.1 ± 84.5 pg/ml LTC: 290.9 ± 83.5 pg/ml	p < 0.001
		Changes of HTC and LTC after overloading period LTC: 304.9 ± 98.9 pg/ml	HTC: 479.2 ± 133.2 pg/ml	p > 0.05
		Changes of HTC and LTC after tapering period	HTC: 508.2 ± 288.4 pg/ml LTC: 334.3 ± 86.0 pg/ml	p > 0.05
Azarbayjani et al. 2011 [21]	IgA	Pre- to post-exercise at week 1	Not provided (Decreased)	p < 0.02
		Pre- to 1-hour post-exercise at week 1		p < 0.01
		Post- to 1-hour post-exercise at week 1		p < 0.01
		Pre- to post-; Pre- to 1-hour post-; Post- to 1-hour post-exercise after 8 weeks of training		p < 0.35
		Changes in resting levels of IgA from Week 1 to Week 2	Week 1: 2530.5 ± 1172.81 ng/ml Week 2: 1320.5 ± 552.38 ng/ml Week 4: 2151 ± 822.99 ng/ml Week 6: 1054.5 ± 443.76 ng/ml Week 8: 587.5 ± 274.65 ng/ml	p < 0.001
		Changes in resting levels of IgA from Week 2 to Week 4		p < 0.001
		Changes in resting levels of IgA from Week 1 to Week 6		p < 0.01
		Changes in resting levels of IgA Week 4 vs. Week 6		p < 0.01
		Comparison in resting levels of IgA at week 8 vs. weeks 1, 2, 4 and 6		Highest reduction p = n/a
	TP	Pre- to post-exercise at week 1	Not provided (Increased)	p < 0.19
		Pre- to post- and pre- to 1-hour post-exercise at week 8		p < 0.01
		Post- to 1-hour post- at week 8		p < 0.01
		Changes in resting levels of TP from week 1 to week 2	Week 1: 5315 ± 1197.05 ng/ml Week 2: 3365 ± 1139.84 ng/ml Week 4: 4705 ± 1027.25 ng/ml Week 6: 2455 ± 992.86 ng/ml Week 8: 1995 ± 451.28 ng/ml	p < 0.05
		Changes in resting levels of TP from week 2 to week 4		p < 0.05
		Comparison in resting levels of TP at week 6 vs. weeks 1, 2 and 4		p < 0.05
		Comparison in resting levels of TP at week 8 vs. weeks 1, 2 and 4		p < 0.05
		Changes in resting levels of TP from week 6 to week 8		p > 0.05
	IgA:TP	Pre- to post-exercise at week 1	Not provided (Decreased)	p < 0.01
		Pre- to 1-hour post- at week 1		p < 0.01
		Post- to 1-hour post- at week 1		p < 0.01
		Pre- to post-exercise at week 8		p < 0.04
		Pre- to 1-hour post- at week 8		p < 0.01
		Post- to 1-hour post- at week 8		p > 0.05
		Comparison in resting levels of TP at week 8 vs. weeks 1, 2 and 4	Week 1: 0.49 ± 0.21 ng/ml Week 2: 0.41 ± 0.15 ng/ml Week 4: 0.47 ± 0.18 ng/ml Week 6: 0.45 ± 0.15 ng/ml Week 8: 0.31 ± 0.15 ng/ml	p < 0.05
Moraes et al. 2017 [22]	IgA	Change of concentration during experimental period	Not provided (Decreased)	p = 0.004
		Pre- to post-intensified training period		p = 0.05
		Comparison of concentrations from pre-experimental period and post-tapering		p = 0.002
		Interaction between low and high aerobic fitness level groups and sampling point	Not provided	p = 0.344
Moreira et al. 2008 [42]	IgA	Pre- to post-17-day preparation training	Pre: 541 ± 226 ug/ml Post: 381 ± 111 ug/ml	p = 0.02
	Secretion rate IgA		Pre: 215 ± 88 ug/ml Post: 188 ± 122 ug/ml	p = 0.30
	TP		Pre: 1.72 ± 0.32 mg/ml Post: 1.58 ± 0.28 mg/ml	p = 0.23
	IgA:TP		Pre: 315 ± 114 ug/ml Post: 243 ± 66 ug/ml	p = 0.04

Note: * – data are reported as mean ± SEM (standard error of the mean); C – cortisol; T – testosterone; T:C – testosterone-to-cortisol ratio; IgA – immunoglobulin A; TP – total protein; LF – lactoferrin; HTC – high testosterone concentration group; LTC – low testosterone concentration group; T4 – week 1 of pre-season training; T1 – week 4 of pre-season training; C1 – week 1 of in-season matches; M1 – week of rest between in-season weeks; C2 – week 2 of in-season matches; R1 – week 1 of post-season recovery; R4 – week 4 of post-season recovery.

Six articles evaluated the effect of periodized training periods on salivary markers [15, 18, 22, 23, 36, 42]. One study examined the C responses in elite female basketball players following a 40-day periodized training period including endurance, strength and power training sessions, finding no changes in C levels across these periods [23]. Similarly, Nunes et al. [18] found no significant changes in salivary C, T or IgA in elite female basketball players after two 3-week overloading periods followed by two taper periods (1 and 2 weeks, respectively). Moreover, in a study assessing the changes of C, T, T:C and IgA during a periodized training period of 50 days including endurance strength and power training, no changes (p > 0.05) were observed over time for C and T levels [15]. In contrast, T:C levels increased in samples collected at 7.30 am compared to pre-training levels, while post-training IgA levels decreased in samples collected at 9.30 am and 11.00 am compared to pre-training levels [15].

The assessment of differences between adolescent basketball players separated into high and low T concentration groups, following baseline measures, resulted in no changes in either group following 5 weeks of overloading and 3 weeks of tapering training periods [36]. The effect of a 17-day preparation period for the Pan American Games including basketball-specific training, sprints, intermittent running exercises and weight training on IgA, TP and their ratio was also examined [42]. The results showed a significant decrease (p = 0.02) in IgA values and the IgA:TP ratio (p = 0.04) from the pre- to post-preparation period, while no difference was found in TP values [42]. In contrast, a significant decrease in IgA levels was observed following an 8-week training period consisting of 1 week of familiarization, 4 weeks of intensified training and 3 weeks of tapering in under-17 male basketball players [22].

Six articles investigated the changes in salivary markers across the basketball season [21, 25, 26, 32, 40, 51], with four assessing youth male basketball players (college and under 19) [25, 26, 32, 40]. He et al. [25] investigated the differences of C, IgA, TP and LF levels collected at different times of the college basketball season including 4 weeks of the pre-season, 3 weeks of the in-season and 4 weeks of the post-season phase compared to those collected at the end of the post-season rest period. Overall results revealed higher C and lower IgA and LF levels during the pre-season and in-season periods compared to values collected at the end of the resting phase, while no significant differences were found for TP values [25]. In a similarly designed study, Andre et al. [26] analysed the weekly fluctuations of C, T and T:C levels across a full season in NCAA Division I college basketball (27 weekly values out of 30 weeks), comparing them with the mean season values. They reported a fluctuating trend across the investigated weeks with the main decrease in C levels at the end of the pre-season and in-season phases, and an increase in the middle of the in-season phase in the week following the holiday break and during the post-season phase [26]. A different trend was observed for T levels, which showed no difference from the mean full-season value during the pre-season phase (weeks 1–6). Conversely, an undulating trend was shown during the in-season (weeks 7–24) and post-season (weeks 25–30) phases with higher values on weeks 13, 15, 17, 28 and lower values on weeks 16, 21, 22, 24, 25, 27 [26]. The analysis of T:C showed higher values compared to the mean value at the end of the pre-season phase (week 7) and lower values in one week during the in-season and post-season phase (weeks 17 and 25, respectively) [26]. When assessing the differences in C values from the pre- to post-in-season phase (~4 months duration) in male Division II college basketball players, a significant increase (p < 0.01) was observed [32]. A further investigation documented an increase in C levels in elite male under-19 basketball players from the beginning to the end of a 4-week period (3 weeks of constant load followed by one week of reduced load) during the in-season phase before the commencement of the playoff phase [40]. On the other hand, no differences were found in IgA absolute values, while a statistically significant decrease (p < 0.05) was observed in the IgA rate value at the end of the studied period [40]. When considering adult players, Gonzalez-Bono et al. [51] analysed changes in salivary C, T and T:C before and after a cycle ergometer test before and after a 4-month period at the beginning of the sport season in two professional male basketball teams exposed to different workloads. The results revealed that the team experiencing lower volume responded differently to the cycle ergometer test with lower pre-test C levels and higher post-test C values compared to the team with higher volume [51]. However, no significant differences were found for T levels across the 2 testing sessions, while the T:C ratio decreased for the team experiencing higher training volume compared to the team experiencing lower training volume [51]. A study evaluating the changes in IgA, TP and IgA:TP ratio across 8 weeks of the pre-season period (data collected at weeks 1, 2, 4, 6 and 8) in male amateur basketball players revealed an undulating trend in IgA and TP values, with higher values collected in weeks 1 and 4 and lower values collected in the remaining weeks [21]. Conversely, IgA:TP ratio showed lower results only in week 8 compared to weeks 1, 2, and 4 [21].

### Salivary markers’ responses to short-term training periods

Three articles assessed changes in salivary markers following a short-term training period [16, 19, 41] (Table 8).

**TABLE 8:** Short-term effect of basketball training periods on salivary marker levels

Study	Marker	Measures	Level (mean ± SD)	Changes
Moreira et al. 2018 [19]	C	Pre-control to post-SSG	Not provided	ES from 0.00 to 0.15 Small and unclear change
		Pre-experimental Stroop to post-SSG		
		Post-control to post-SSG		
		Post-experimental Stroop to post-SSG		
	T	Pre-control to post-SSG	Not provided	ES (90% CI) = 0.98 (0.42;1.50) Large and clear increase
		Pre-experimental Stroop to post-SSG		ES (90% CI) = 0.33 (-0.18; 0.83) Small-to-moderate difference
		Post-control to post-SSG		ES (90% CI) = 0.66 (0.13;1.16) Moderate and clear increase
		Post-experimental Stroop to post-SSG		ES (90% CI) = 0.37 (-0.14;0.86) Small-to-moderate and unclear change
	AA	Pre-control to post-SSG	Not provided	ES (90% CI) = 0.82 (0.22; 1.38) Large and clear increase
		Pre-experimental Stroop to post-SSG		ES (90% CI) = 0.55 (0.00;1.10) Moderate increase
		Post-control to post-SSG		ES (90% CI) = 0.44 (-0.12;0.99) Moderate and unclear increase
		Post-experimental Stroop to post-SSG		ES (90% CI) = 0.13 (-0.40;0.67) Small and unclear difference
Nunes et al. 2011(b) [41]	C	Comparison of non-exercising day (NE) and post-endurance training scheme (ES)	Not provided (Increased)	p < 0.05
		Comparison of NE and strength-hypertrophy training scheme (SHS)		p < 0.05
		Comparison of NE and power training scheme (PS)		p < 0.05
		Pre- to post-SHS		p < 0.05
		Comparison of post-SHS and post-ES and post-PS		p < 0.08
	T	Pre- to post-endurance (ES), strength-hypertrophy (SHS) and power (PS) schemes	Not provided	p > 0.05
		Comparison of ES, SHS and PS with levels from non-exercising day (NE)		p > 0.05
		Time effect (7:30, pre, post and 17:30) on levels of testosterone during ES, SHS, PS and NE days.		p > 0.05
	IgA	Pre- to post-endurance (ES), strength-hypertrophy (SHS) and power (PS) schemes	Not provided	p > 0.05
		Comparison of ES, SHS and PS with levels from non-exercising day (NE)		p > 0.05
		Time effect (7:30, pre, post and 17:30) during ES, SHS, PS and NE days		p > 0.05
Sansone et al. 2018 [16]	C	Pre-small-sided game concentrations	Off-long: 6.7 ± 4.7 ng/ml Off-short: 7.3 ± 2.2 ng/ml D-long: 8.1 ± 4.3 ng/ml D-short: 6.6 ± 1.4 ng/ml	p = 0.599
		Interaction between time, task and regime	Not provided	p = 0.350
		Interaction between task and regime		p = 0.295
		Interaction between task and time		p = 0.485
		Interaction between regime and time		p = 0.757
		Interaction for time		p = 0.001
		Interaction for task		p = 0.694
		Interaction for regime		p = 0.128
	T	Pre-small-sided game concentrations	Pre-off-long: 200.7 ± 86.7 pg/ml Pre-off-short: 260.5 ± 155.9 pg/ml Pre-D-long: 159.3 ± 94.7 pg/ml Pre-D-short: 175.1 ± 183.1 pg/ml Post-off-long: 239.4 ± 122.2 pg/ml Post-off-short: 192.2 ± 152.9 pg/ml Post-D-long: 251.8 ± 104.3 pg/ml Post-D-short: 249.0 ± 130.22 pg/ml	p = 0.227
		Comparison of post-small-sided games concentrations		p > 0.05
		Pre- to post-offensive-long		p > 0.05
		Pre- to post-offensive-short		p = 0.028
		Pre- to post-defensive-long		p = 0.037
		Pre- to post-defensive-short		p > 0.05

Note: C – cortisol, T – testosterone; AA – alpha-amylase; IgA – immunoglobulin A; NE – non-exercising day; ES – muscle endurance training scheme; SHS – strength-hypertrophy training scheme; PS – power training scheme; Off-long – long-intermittent training regime with offensive task; Off-short – short-intermittent training regime with offensive task; D-long – long-intermittent training regime with defensive task; D-short – short-intermittent training regime with defensive task.

Acute responses in C, T and IgA values to muscle endurance, strength-hypertrophy and power training were compared to values collected during resting days [41]. The main results showed higher levels of C in each examined training typology compared to resting days, with strength-hypertrophy training eliciting higher C secretion compared to pre-training values [41]. Conversely, no significant differences were found for T and IgA across the three studied training modalities [41]. One article examined the acute effect of 3 × 3 basketball small-sided games (SSGs) played with different tactical tasks (offense vs. defence) and training regimes (long vs. short) on C, T values [16]. No significant interactions were found between the three investigated independent variables [i.e. time (pre- vs. post-SSG), task and regime] for C levels with effect sizes ranging from no effect to minimum [16]. When considering the independent variables separately, a time effect was found with a significant increase in C level in post-SSG compared to pre-SSG values with a strong effect size, while no significant differences were found for task and regime [16]. When considering T values, a decrease in T concentration was found at the end of the SSGs combining a short regime and an offensive task (moderate effect size) and an increase in those combining a long regime and a defensive task (moderate effect size) when compared with values collected before SSGs [16]. Overall, no significant differences were found when comparing the T values collected at the end of each SSG [16]. In a unique study, Moreira et al. [19] investigated the effects of mental fatigue in comparison to a control group on C, T, AA (pre- vs. post-condition) and the responses of these markers following a subsequently played 4 × 4 SSG (post-SSG). Small (ES = 0.0–0.15) and unclear changes were found in C concentration within both conditions [19]. By contrast, T concentration greatly increased from pre-control condition to post-SSG (ES = 0.98; 90%CI = 0.42–1.50), moderately increased from post-control condition to post-SSG (ES = 0.66; 90%CI = 0.13–1.16), while unclear changes were observed from post-mental fatiguing condition to post-SSG (ES = 0.37; 90%CI = -0.14–0.86) [19]. Considering AA concentrations, values greatly and moderately increased from pre-conditions to post-SSG for control and mental fatiguing conditions, respectively [19].

## DISCUSSION

### Reliability of results

Higher reliability of results indicates high precision of measurements with the coefficient of variation as one of the most useful calculations adopted for this analysis [52]. Specifically, for the assessment of salivary hormones, acceptable reliability is considered when the coefficient of variation for intra- and inter-assays is lower than 10% [53]. The results of this systematic review indicate that the reliability values of included papers were reported for twelve out of fifteen articles with coefficient of variation values < 10% (Table 6). However, there are three included articles with no coefficient of variation values reported, which indicates that the results might be inaccurate [21, 40, 42]. Nevertheless, these three manuscripts not reporting the coefficient of variation for the intra- and inter-essay documented a similar score in our assessment of methodological quality compared to other included papers (Table 4).

### Long-term training periods

The twelve included articles considering the effect of long-term training periods on salivary markers mainly focus on the assessment of periodized training periods [15, 18, 22, 23, 36, 42], and the basketball season [21, 25, 26, 32, 40, 51]. Additionally, different basketball populations were investigated in the reviewed papers and specifically: i) amateur youth [22, 36] and senior male players [21]; ii) sub-elite collegiate male players [25, 32]; iii) elite youth [40], collegiate [26] and senior male players [42, 51]; iv) elite senior female players [15, 18, 23]. Due to the different long-term period typologies, playing levels, age and gender, the findings showed inconsistent responses of the different investigated salivary markers (C, T, T:C, IgA, TP, LF).

### Cortisol

Salivary C was found unresponsive to three differently designed periodized training programmes in elite female basketball players [15, 18, 23]. The unresponsiveness of C might be explained by the fact that, although the studied periodized training programmes involved a high workload, they were lacking official competitions [15, 18, 23]. In fact, a previous study assessing the serum C level changes during the pre-season and in-season phases across 4 seasons in elite male basketball players demonstrated that although players experienced a higher workload during pre-season, the in-season phase stimulated higher serum C levels due to the physiological and psychological stress induced by official matches [10].

When considering the C changes across the basketball season, it is hard to make any comparison across the reviewed studies since different time periods, frequency of saliva sampling, basketball populations and study designs were investigated [25, 26, 32, 40, 51]. Mostly, these studies assessed the changes in C levels during the in-season phase and either in comparison with other season phases [25], or within the in-season phase monitored entirely with weekly measures [26]; verified the differences from pre- to post-in-season phase [32]; and monitored the partial in-season phase [40]. Considering the differences between phases in the basketball season, higher C levels were observed during the in-season and pre-season phases due to higher physical stress imposed by the training and matches compared to the post-season recovery period [25]. When considering the changes in C levels within the in-season period, Atalag et al. [32] found an increase in C levels across the in-season phase in Second Division college basketball players. This outcome might have been influenced by the frequent air travels in different time zones throughout the course of the season, which might have an influence on players’ sleeping patterns and consequently on the circadian cycle of C [54]. In a rare study assessing the changes in C levels across the in-season phase using a more frequent monitoring approach (weekly changes), several weekly fluctuations were found in comparison with the average C value measured across the studied phase in collegiate male basketball players [26]. However, these results might have been more informative when indicating the weekly fluctuations in C levels rather than in comparison with the average seasonal value, therefore indicating the necessity of a more appropriate study design and statistical analysis approach in future investigations. Only one of the reviewed manuscripts assessed the changes in C levels in a part of the in-season phase and specifically during the last 4 weeks before the commencement of the playoffs, indicating an increase in C levels [40]. The increase in C levels was found concomitantly with a reduction in training load during the investigated period, indicating that other factors (e.g. psychological, lifestyle) rather than training volume might be responsible for these changes.

The changes of C levels during the basketball season were also measured jointly with training load [51]. It was found that adopting different training loads in two elite male basketball teams during a 4-month season period led to different changes in C concentration in response to a cycle ergometer test [51]. Specifically, it was found that the team experiencing lower volume responded with significantly lower pre-test C levels and higher post-test C values compared to the team with higher volume [51]. This different responsiveness confirms that experiencing a higher workload has a positive effect on adaptive levels of the hypothalamic-pituitary-adrenal axis, resulting in lower sensitivity of C concentration [23, 55]. However, it should be noted that these results assessed C levels only in response to the cycle ergometer test, while a more isolated measure of the C levels in a properly designed study would provide a better indication of the C responses to different training loads in basketball players.

### Testosterone

Considering the response of salivary T in elite female basketball players, similar responses to those found in C levels were reported, showing no changes in T concentration following a 50-day periodized training [15] or 12-week periodized preparation including 2 overloading periods [18]. These results are in line with a previous study assessing the differences in T levels over a 12-week training and competitive period in female athletes from different sports (i.e. track and field, cycling, swimming and bob skeleton) showing no differences in T levels from the beginning to the end of the investigated period [56]. These outcomes might indicate the low responsiveness of T to training stimuli over long-term training periods in female athletes. However, it should be noted that, when T changes were monitored with higher frequency (i.e. weekly), significant fluctuations were found in female athletes from different individual sports [56], highlighting the importance of monitoring T to assess the internal response to training stimuli. In fact, the different overloading and taper periods might play a role in detecting no changes in T levels over long-term training periods, and monitoring T with a higher frequency might provide more detailed information of the T changes due to the imposed stimulus.

It should be noted that no changes in T concentration were also found in two groups of youth basketball male players following overloading and tapering periods [36] and in two elite male teams experiencing different workloads during a 4-month period during the basketball season [51]. A possible explanation of T unresponsiveness during long-term training might be due to the load and recovery experienced by the investigated players. Indeed, the amount of load and recovery might not have led to overtraining conditions, which would have induced a decrease in T concentration, as suggested by Coutts et al. [57] when investigating rugby league players during a 7-week sport-specific preparation period. Moreover, the experienced load might not have been appropriate to induce an increase in T levels, which would be expected as an anabolic response to the applied training stimulus and recovery.

When considering weekly changes in T levels across an entire season in male college basketball players, a fluctuating trend in T responses was observed, with higher levels found compared to the season mean value after recovery periods and before regular season matches, while at the end of the season and before away and playoff matches T concentration was below the season mean [26]. Higher T levels after recovery can be explained by higher activity of the hypothalamic-pituitary-gonadal axis to induce greater anabolic and anti-catabolic processes involved in muscle tissue growth, physical and physiological recovery and remodelling for performance enhancement [58, 59]. Considering higher concentration of T before important in-season matches, an increase can be explained by higher readiness to compete against opponents and overcome psychological threats to lose, promoted by increased stress levels [25, 63]. Moreover, lower T levels were found after travelling to play an away match and before the beginning of the playoff phase [26], which can be explained by reduced self-confidence and higher perceived threats before these periods [25, 63]. Another possible factor contributing to lower T levels at the end of the season and before playoff matches is a detrimental physiological effect of a long season on collegiate basketball players [26]. Indeed, accumulative physiological and psychological exertion with a huge increase in stress levels before playoff matches probably inhibited the release of T concentration [62, 63].

### Testosterone-to-cortisol ratio

Salivary T:C is considered one of the main markers indicating an adaptive response to training [30]. T:C is documented as a marker including both anabolic and catabolic processes and therefore is sensitive to the applied training volume and physiological stress [30]. Adrenocorticotrophic hormone (ACTH) has been reported to have a dominant role in T:C changes, as secretion of corticotropin hormone in response to physiological stress stimulates the release of ACTH from the anterior pituitary, which in turn stimulates the release of C from the adrenal cortex, resulting in a decrease of T:C levels, leading to reduced adaptive processes [60, 61].

In this systematic review, three manuscripts assessed the changes in T:C levels during a long-term training period [15, 26, 51]. Nunes et al. [15] found no changes in T:C levels as well as T and C levels during 50-day periodized training consisting of muscle endurance, strength-hypertrophy and power training periods in elite female basketball players. This indicator followed the results obtained in C and T levels during long-term periodized training. However, in the same study, significant changes in T:C were evident when considering measures collected at different times of the day, showing higher T:C from samples collected at 7:30 am after the training programme, compared to the pre-training value [15]. In contrast, no effect for the time of the day was found for T and C levels, suggesting that T:C ratio is more sensitive to minor changes than the markers separately and might be a superior indicator of adaptive levels [30].

When considering the weekly T:C changes across an entire basketball season, an investigation in elite collegiate basketball players showed T:C values to be different in 3 weeks compared to the 30-week season mean value [26]. Firstly, higher T:C values were found at the beginning of the regular season, showing an advantage of the tapering period performed at the end of the pre-season phase as adaptive levels increased above the season mean value [26]. In agreement with findings in American football [62] and elite track and field athletes [63], a tapering period at the end of preparation has an effective impact for super-compensation on the balance between anabolic and catabolic processes. However, T:C was below the season mean before the beginning of important in-season matches and before playoff matches [26]. The difference in T:C levels during these weeks suggests that the perceived threat of upcoming important matches and the accumulated physical, physiological and psychological stress at the end of the season might have diminished the activity of the hypothalamic-pituitary-gonadal axis, resulting in lower adaptive levels [60].

In another investigation [51], T:C changes were assessed before and after 4 months of the basketball season in two elite male teams, with their season training volume being different by two-fold [51]. The results revealed a decrease of T:C values for the team experiencing higher workload and an increase in T:C values for the team with lower workload [51]. This result indicating a possible negative impact of workload on the T:C values was also corroborated by the inverse relationship between T:C and workload [51]. Indeed, due to accumulative psychophysiological stress, constantly applied high workload reduces adaptation to training, while lower workload does not cause inhibition of hypothalamic-pituitary-gonadal axis activity or reduced adaptive levels [26, 51, 60, 64].

### Immunoglobulin A

Salivary IgA is considered as a potential marker for determination of excessive training, psychological stress and wellness of the upper respiratory tract [22, 65]. The main function of IgA is to stop viral infections and to inhibit the attachment of bacteria and viruses at the mucosal epithelium in the upper respiratory tract [22]. However, due to the excessive workload, production of IgA can be suppressed, resulting in higher risk of URTI [66].

Five included articles reported the IgA response to different training programmes (i.e. periodized training, overloading and tapering periods, preparation for the season) in different basketball populations [15, 18, 21, 22, 42]. In particular, a reduction of IgA levels was found across: i) a 50-day periodized training period in elite female players [15], ii) 8 weeks of continuous and intermittent training in amateur male players [21], and iii) a 17-day preparation period in elite male players [42]. Nevertheless, one study reported no changes in IgA concentration after a 12-week training period including two overloading and tapering phases in elite female basketball players [18]. This difference in the results might be attributed to the use of a tapering phase in the assessed training periods. Indeed, a decrease in IgA levels following training without a tapering phase might result in an excessive workload and high psychophysiological stress, causing suppression of IgA production [15, 21, 42], while the use of tapering periods could contribute to the reduced negative effect of high stress on mucosal immunity [18]. While these studies focused on senior basketball players [15, 18, 21, 42], different outcomes were obtained in youth male basketball players. In fact, Moraes et al. [22] reported a significant reduction in IgA levels following both a 4-week intensified training period and the subsequent 3-week tapering phase. This reduction in IgA levels can be explained by lower tolerance to high physiological and psychological stress in youth players compared to senior players [40]. Indeed, an increase in psychophysiological stress can lead to a reduction of IgA levels due to the altered functions of immune cells mediated by stress hormones [67].

Two papers further assessed the changes in IgA values following long-term training periods across the basketball season [25, 40]. He et al. [25] recorded lower IgA levels during pre-season and in-season phases in sub-elite collegiate male basketball players compared to the values registered after a 4-week post-season recovery period. These outcomes confirm that the pre- and in-season phases cause deterioration of mucosal immunity function due to psychophysiological stress induced by high training load and official matches [24]. This result is also corroborated by a previous study assessing the changes IgA and C levels in elite youth players during 4 weeks of the in-season phase before the beginning of the playoff phase [40]. While a reduction of the training load during occurred in the last investigated week, no significant changes in IgA levels were evident across the entire 4-week period, possibly due to the high stress levels experienced by players in this important phase of the in-season [40]. Indeed, an increase in C secretion was reported, which in turn suppressed the release of IgA [40]. Overall, the findings show that during long-term training periods, the mucosal immunity system can be negatively affected, and consequently lead to a reduction of IgA secretion, due to high psychophysiological stress induced by the preparation and competitive phases of the season.

### Other salivary markers

The effect of long-term training periods was also investigated on other salivary markers such as TP and IgA:TP in amateur male [21] and elite male basketball players [42], while changes in TP and LF were also examined across an entire basketball season in sub-elite collegiate male basketball players [25].

Salivary TP is considered as one of the main markers representative of players’ hydration status [68, 69]. Azarbayjani et al. [21] reported a progressive decrease in TP levels during 8 weeks of continuous and intermittent training periods, designed with gradual reduction of rest time during exercise with the work-to-rest ratio changing from 1:4 to 1:1, resulting in increased training intensity. However, when considering the long-term effect on TP levels during a basketball season, no changes in absolute concentration or secretion rate were found in sub-elite collegiate male basketball players [25]. Players’ hydration status plays a fundamental role in the secretion of salivary TP [68, 69]; therefore, the contrasting results of these investigations might be attributed to the different amount of fluids consumed by the investigated players during the investigated periods. Indeed, the loss of the whole body fluids and a long time for their recovery can inhibit the activity of SNS, which is responsible for production and release of TP [68–70]. When considering the effect of a 17-day preparation period for the Pan American Games on TP levels, no significant changes were found [42]. However, the results of this study should be considered with caution since TP responses were investigated in five elite male basketball players and with five staff members with combined results reported and therefore not allowing a proper understanding of the effect of the preparation periods on players’ TP levels [42].

The high psychophysiological stress and reduced immune function during important phases of the basketball season or during long-term preparation periods have also been demonstrated via the analysis of further salivary markers such as IgA:TP [21, 42] and LF [25]. A significant decrease in IgA:TP was documented for amateur [21] and elite [42] male basketball players following long-term training periods. Formerly, the IgA:TP has been suggested as a marker showing a more evident effect of physiological and psychological stress on the immune system [71]; however, recent research [72, 73] showed that TP secretion rate can increase due to exercise or any other stimuli for SNS, leading to disturbed IgA:TP and suggesting caution in the interpretations of these results. Additionally, LF is considered as a marker of innate mucosal immunity, with a previous study showing a detrimental effect of high training and match loads experienced during the pre- and in-season phases on immune function in sub-elite collegiate basketball players [25]. Overall, these outcomes confirm that long-term training periods have a negative effect on the mucosal immunity due to the high physiological and psychological stress.

### Short-term training periods

The findings of this review show that only three studies investigated the effect of short-term training periods on salivary markers in basketball [16, 19, 41].

#### Cortisol

Overall, inconsistent results were obtained for the effect of short-training periods on C levels due to different training typologies (endurance, strength, power training and SSGs) and sample characteristics (sex and age categories) [16, 19, 41]. Nunes et al. [41] reported that one session of strength-hypertrophy training increased C levels compared to pre-test, a non-exercising day, one session of power training and one session of endurance training in female basketball players. A possible explanation of these findings is that post-exercise C values are influenced by the total volume of the training session, which was higher in the strength-hypertrophy training session compared to other training sessions [41]. Indeed, it was stated that C is the predominant catabolic hormone that regulates a decrease of protein synthesis and increase in protein breakdown during exercise to induce higher use of amino acids for energy production [60, 74]. Possibly, the strength-hypertrophy scheme, due to having the highest training volume, induced higher use of amino acids in comparison with muscle endurance and power sessions [41].

When considering the effect of SSGs on C levels, the results were also inconsistent. Sansone et al. [16] reported that 12 min of half-court SSGs played 3 × 3 and with different tasks (defence vs. offense) and regime (long-intermittent vs. short-intermittent) strongly enhanced the C levels compared to pre-SSG values in male semi-professional basketball players. Conversely, Moreira et al. [19] observed small, unclear changes in C level following 10 min SSGs played 4 × 4 while comparing control and mental fatigue conditions in male elite youth basketball players. As previously stated, C might be influenced by the total workload elicited by the proposed training sessions. However, it is not possible to make a comparison between the workload elicited by the SSG typologies proposed in the study of Sansone and Moreira [16, 19]. Nevertheless, it seems that the SSG modality investigated in Sansone’s manuscript [16] might have elicited a higher training stimulus and stressful condition similar to those reported in official matches [39] compared to the SSGs studied in Moreira’s paper [19]. These inconsistencies in the results call for further analysis in comparing the effect of SSGs on C levels when playing with different modalities and in relation to the workload elicited.

#### Testosterone

Unlike the results for C, no effect was observed from pre- to post-training for T levels across one-day training sessions of strength-hypertrophy, power or endurance in female basketball players [41]. This lack of changes might be due to the non-prominent role of T in female athletes compared to growth hormone, dehydroepiandrosterone and oestradiol, which might have a more important anabolic role during and after resistance training [1, 29, 41, 55]. Therefore, female T level is essentially unresponsive to these kinds of resistance training modalities. However, it should be noted that contrasting results were found in the literature about the acute effect of resistance training on T levels in female athletes [75], and considering that only one study was found in the literature on female basketball players, more research is warranted.

In contrast to female athletes, T is one of the main anabolic markers in male athletes [76, 77]. In fact, male and female athletes have been shown to respond differently when using the same relative load [76, 77]. Therefore, it would be worthwhile to assess whether different resistance training modalities could have an impact on T levels in male basketball players, calling for further studies.

In our review, the effect of short-term training basketball periods was reported in male semi-professional [16] and youth athletes [19] only in the form of SSGs, with the results highlighting contrasting outcomes of T levels following differently-designed SSGs. In fact, Sansone et al. [16] documented a decrease in T levels in SSGs played with an offensive task and a short regime, which elicited a high training volume measured via microsensors (i.e. PlayerLoad = ~152 AU). Contrarily, SSGs played with a defensive task and long regime inducing a lower volume (PlayerLoad = ~133 AU) showed an increase of T levels in semi-professional players [16]. Moreover, Moreira et al. [19] comparing control and mental fatigue conditions playing a 4 × 4 SSG found a moderate increase in T levels at the end of the SSG in the control condition, while a small increase was found in mentally fatigued players. Previous research mainly focused on analysing the effect of resistance training on T levels [75], rather than game-based activities. Overall, due to the activation of the central nervous system (CNS), an increase of T levels following resistance training with an appropriate volume, intensity and recovery was observed as an expected response [75]. The activated CNS innervates the hypothalamus, which provides a direct link between the nervous and endocrine systems, allowing a quick delivery of the hormonal signal to the pituitary target cells, where gonadotrophin releasing hormone (GnRH) stimulates the production of luteinizing hormone (LH) and follicle-stimulating hormone (FSH) from gonadotrophs [75, 78]. Produced LH and FSH then enter the circulation and are transferred to the gonads, where LH stimulates the production of T, which is subsequently released [75]. However, even though there is more scientific background for T production and release following different types of training modalities, no other studies have assessed the neuro-physiological mechanisms involved in SSGs in team sports and specifically in basketball, calling for further investigations.

#### Immunoglobulin A

Similarly to the results for T, no changes or differences were found for IgA concentration following muscle endurance, power and strength-hypertrophy resistance training schemes in elite female basketball players [41]. This is the only study assessing changes in IgA values in basketball players following short-term training periods, making comparison with other studies not possible. Previous literature indicated that prolonged strenuous exercise might induce a reduction in IgA values, which is associated with an increased frequency of URTI episodes [79]. Therefore, the schemes proposed by Nunes et al. [41] might have induced a training stimulus not sufficient to induce changes in IgA values, similar to the outcomes documented in untrained old women performing two strength training schemes [80], and in trained and untrained women following a strength workout [81]. The unresponsiveness of IgA levels in female players following specifically designed training schemes calls for a further investigation with training modalities inducing different workloads.

### Other salivary markers

The only salivary marker assessed in response to short-term training periods, and specifically to SSGs, is AA [19], which indicates the activity of the sympathetic nervous system (SNS) as a response to physical and physiological stress [82]. Moreira et al. [19] assessed the AA responses to SSGs in control and mentally fatigued groups, finding a large increase from pre- to post-SSG values in the control group and a moderate increase from pre- to post-SSG values in the mentally fatigued group. It was suggested that the stress induced by the activity performed during SSGs would increase players’ stress levels due to the possible elevation in SNS activity and consequently the AA values [19]. Alternatively, the mental fatigue condition might have compromised the activity of the SNS and therefore led to lower production of AA [19]. Overall, this study is unique in assessing the effect of short-term training periods in the form of SSG and according to control and mental fatigue conditions, indicating the need of further studies in assessing the short-term effect of different training typologies on AA responses.

## CONCLUSIONS

This review was the first to provide a systematic evaluation of the changes in salivary markers in response to long- and short-term training periods in basketball. Regarding long-term training periods with different durations, the main findings indicate no changes in different basketball player populations during long-term periodized training periods in C, T and their ratio, while contrasting results were found in IgA and TP levels and a reduction in IgA:TP levels. When considering changes in salivary markers thorough different phases of the season, a tendency of higher C and lower IgA levels during pre-season and in-season phases compared to the post-season recovery phase was observed, and weekly fluctuations were observed for T, C and T:C. The analysis of the effect through short-term training periods on salivary markers documented that strength-hypertrophy training induced higher C levels compared to a non-exercising day, one-power training and one-endurance training session in female basketball players while no changes were evident for T and IgA. Moreover, the analysis of salivary markers in response to SSGs documented a large-to-moderate increase in AA from pre- to post-SSG and inconsistent results of C and T changes across differently designed SSGs.

## PRACTICAL APPLICATIONS

There is a limited number of studies focusing on the assessment of salivary markers in basketball. However, the analysis of salivary markers in combination with other measures assessing the internal responses to stimuli could provide a more detailed analysis of the players’ physical fitness status and well-being in response to basketball training. Therefore, we would suggest to basketball practitioners and sport scientists the use of salivary markers and the development of future studies assessing changes in salivary markers in basketball. In particular, future studies should overcome the limitations of the studies included in this systematic review and in particular adopt i) more appropriate study designs, ii) more robust statistical analysis with the inclusion of the effect sizes providing a practical interpretation for the changes, iii) multiple-team studies, which could provide more robust sample sizes, iv) the analysis of salivary markers’ response in conjunction with internal and external load measures and well-being questionnaires, v) a comparison of salivary markers’ responses between female and male basketball players, vi) more frequent sampling of saliva collection during the investigated training periods to have a better understanding of the salivary marker fluctuations.

## Funding

This research did not receive any specific grant from funding agencies in the public, commercial, or not-for-profit sectors.

## Conflict of interest

The authors declare that they have no conflict of interest with the content of this systematic review.

## Author contributions

Paulius Kamarauskas 50%; Daniele Conte 50%.
